# Open science at the science–policy interface: bringing in the evidence?

**DOI:** 10.1186/s12961-022-00867-6

**Published:** 2022-06-20

**Authors:** Stefan Reichmann, Bernhard Wieser

**Affiliations:** TU Graz, Graz, Austria

**Keywords:** Research uptake, Evidence–policy gap, Policy theory, Open science

## Abstract

Part of the current enthusiasm about open science stems from its promises to reform scientific practice in service of the common good, to ensure that scientific outputs will be found and reused more easily, and to enhance scientific impact on policy and society. With this article, we question this optimism by analysing the potential for open science practices to enhance research uptake at the science–policy interface. Science advice is critical to help policy-makers make informed decisions. Likewise, some interpretations of open science hold that making research processes and outputs more transparent and accessible will also enhance the uptake of results by policy and society at large. However, we argue that this hope is based on an unjustifiably simplistic understanding of the science–policy interface that leaves key terms (“impact”, “uptake”) undefined. We show that this understanding—based upon linear models of research uptake—likewise grounds the influential “evidence–policy gap” diagnosis which holds that to improve research uptake, communication and interaction between researchers and policy-makers need to be improved. The overall normative stance of both discussions has sidelined empirical description of the science–policy interface, ignoring questions about the underlying differences between the policy domain and academia. Importantly, both open science and literature on closing the evidence–policy gap recommend improving communication (in terms of either the content or the means) as a viable strategy. To correct some of these views, we combine insights from policy theory with a narrative review of the literature on the evidence–policy gap in the health domain and find that removing barriers to access by itself will not be enough to foster research uptake.

## Introduction: the role of science in policy-making

Whereas in earlier decades, public servants played a dominant role in policy advice, they are now expected to consult external sources (academia, stakeholder organizations, think tanks, political organizations) [42]. At least in industrialized countries, governments increasingly rely on external advice and (scientific) evidence as a way to improve governance [44]. This has led to increased interest in understanding the interlinkages between policy-making and expertise [56]. The goal of research in government is to find information that will help to solve specific, predefined policy problems in real time [31]. While the evidence-based policy (EBP) movement was instrumental in promoting rigorous analysis of policy options and programmes [43], a salient issue in the literature concerns the fact that topical scientific expertise is not being used in respective policy decisions despite its availability [36, 39], a problem that has been labelled the “evidence–policy gap” [39] and has been singled out as a major obstacle to reaching the United Nations’s Sustainable Development Goals in public health [69].

In this paper, we investigate literature addressing processes of knowledge integration in the policy domain and thereby focus on the recognition of open science as an effective way to enhance the uptake of scientific knowledge in the policy domain. In fact, utilization of publicly available scientific results and data by policy-makers has been claimed to be one of the benefits of open science [63, 82]. Likewise, a variety of measures have been proposed for researchers who wish to make an impact on policy-makers. Like advocates of open science, analysts of the evidence–policy gap recommend improving communication and other forms of interaction between researchers and policy-makers to close the gap. We argue that inquiring into the role of open science in policy-making can be regarded as a variant of the more general issue of research utilization or research uptake in policy-making. Open science thereby acts as a lens through which the science–policy interface can be studied by asking: How would scientific policy advice be improved if we improved scholarly communication? To this end, we reviewed literature studying the ways in which knowledge enters the policy process as well as literature documenting the reforms to scholarly communication and research practice envisioned by open science.

To the best of our knowledge, science advice has been most comprehensively problematized in the health policy domain, under the heading of an evidence–policy gap [7, 10, 14, 17, 21, 35, 40, 65]. This literature identifies concrete barriers to research utilization, offering suggestions to researchers how those barriers could be overcome. Yet, the relationship between policy-makers and researchers at the science–policy interface is rarely scrutinized and is frequently described as one of mutual misunderstanding or outright mistrust [41], where researchers and policy-makers are incapable of successful communication.

We begin our paper with a brief exposition of the development and tenets of the open science movement. Then we briefly review the history of the science–policy interface to interrogate the potential for open science practices to enhance scientific policy advice. Based on a narrative literature review, we describe how it frames the science–policy relationship, how it suggests the uptake of scientific knowledge in the policy domain can be enhanced and how the (potential) contribution of open science is assessed. Before concluding, we introduce three analytical points to conceptually inform the interpretation of our findings. This work is based upon and significantly extends a project deliverable prepared for the European Commission under Grant Agreement No. 824612 (ON-MERRIT—Observing and Negating Matthew Effects in Responsible Research and Innovation Transition) [73]. For the deliverable, a systematic literature review was performed in Scopus and Web of Science. Articles were selected based on abstract and keywords, with additional hand selection of articles from the sample so generated based on references to (aspects of) open science. Results were then summarized, validated and synthesized. The relevant literature was restricted to (1) all (peer-reviewed) materials pertaining to policy-making, and (2) all materials referring to (aspects of) open science within that corpus. For the analysis, a triaging strategy was used (similar to the approach described in Contandriopoulos et al. [16]) to keep the amount of text manageable. The obtained data are narrative (i.e. in the form of published research articles), so the primary analysis strategy was narrative as well. Combining a summative approach and an analytical approach, we produced a synthesis document of how the literature describes research uptake. We included a total of 115 articles in the review.

## Conceptual background

### A brief exposition of open science

The term “open science” encompasses a variety of meanings ranging from publicizing research outputs—open access in its various forms—to making all aspects of the research process accessible [26], including data (e.g. [30]), notebooks, analysis plans and code [46, 72], as well as research evaluation and peer review [75, 79]. Open science denotes a bundle of practices and associated ideas such as accessibility, reproducibility, (data) sharing and collaboration [84], but is often used interchangeably with open access and open data [2]. With respect to the science–policy interface, some ([82], see also the Budapest Open Access Initiative of 2002 [63]) have suggested that open access facilitates knowledge utilization in policy-making.

In light of the plurality of these approaches, open science is best described as an umbrella term for a programme of reforming science by reforming scholarly communication, with a clearly normative thrust (i.e. to make science better). Proponents have taken for granted the premise that academia is in need of top-down reform, a claim that has been couched in the superficially positive terminology of “openness”, without clearly stating in what way science was ever “closed”. In an attempt to cluster the existing landscape of open science interpretations, Fecher and Friesike described the various approaches in terms of five “schools”: “The *infrastructure school* (which is concerned with the technological architecture), the *public school* (which is concerned with the participation in knowledge creation), the *measurement school* (which is concerned with alternative impact measurement), the *democratic school* (which is concerned with access to knowledge) and the *pragmatic school* (which is concerned with collaborative research)” [25]). Following this framework, we argue that dominant interpretations of open science concern science-to-science interaction and collaboration, while only parts of what sails under the banner of open science addresses science-to-public relationships and engagement. In essence, the “public school” asks questions as to how science can be open to collaboration by the public (for the most part understood as citizen science) and how science can benefit the public.^1^ Open science advocates have suggested that the removal of access barriers will serve to boost public trust in science as well as evidence-based policy-making, with particular emphasis on enrolling the public [37]. Others [81] argue, in the same vein, that the trend towards increased openness holds the promise for more public engagement. Some have claimed that open science continues a long-standing agenda of fostering participatory research. Civil society actors, users, patients, nongovernmental organizations, industry and other societal stakeholders are not only said to benefit from open access to scientific outputs [82] but, crucially, are regarded as resourceful contributors to processes of knowledge production. Therefore, citizen science is highly valued in open science as furthering public engagement throughout, contributing to data collection and analysis and publication and evaluation of research findings [84], as well as facilitating dialogue between science and society [52].

In the next section, we wish to prepare our main point—that both open science and policy advice frequently fall short of delivering adequate empirical descriptions, in particular as regards the science–policy interface—by describing how theorizing the science–policy interface has developed in the second half of the twentieth century.

### Three phases of the science–policy interface

The way in which science and research engage with government underwent a considerable evolution in the second half of the twentieth century [73]. As the science–policy interface evolved, so did conceptual models developed by social and political scientists [32]. Before the Second World War, governments were entrusted with defining the common good. Postwar Europe saw increasing numbers of scholars engaging with policy in an era of increased investment in science [80]. Maasen and Weingart [57] identify several historical developments conducive to the changing relationship between science and policy-making. For the United States, Weingart [85, 86] diagnosed increasing formalization of communication between science and policy that he traced to President Dwight Eisenhower’s 1957 appointment of a science and technology advisor and committee. This model still exists, at least in part [32], but has since been supplemented by more sophisticated models of science advice (e.g. Mode 2 Science, cf. [29]).

In a recent review, Sokolovska et al. [80] describe three broad historical phases the science–policy interface underwent in the second half of the twentieth century, each with its associated policy model: The first phase (“linear models”) corresponds to a linear understanding of the science–policy interface, where advice is conceived as a one-way communication process [80] based on a dichotomy of facts (science) and values (policy). The linear model assumes knowledge integration working rationally from problem identification to problem solution, and assumes that appeals to scientific data and established facts will lead to better problem characterizations and, by extension, better policy. Meanwhile, this linear approach to the science–policy interface has come under criticism. It is now understood that the linear model does not adequately reflect the complexity of the science–policy interface [8] and the processes by which knowledge enters the policy sphere [47]. The second phase (“interactive models”) is characterized by the assumption that both scientists and policy-makers collaborate in a nonhierarchical way in search of the best problem solutions. With respect to the science–policy interface, the role of communicating the results of evidence synthesis falls to the knowledge broker [32], as described in Roger Pielke’s book *The honest broker* [70]. The hitherto final phase (“participatory models”) revolves around the question of engaging society at large in the policy process. Participatory models regard the science–policy interface as a discursive process between researchers and policy-makers [80] and are similar to interactive models in this respect; however, they are characterized by intense reflection on “formats of societal engagement and the language of communication on the science–policy interface” [80], for example, in terms of changing relationships between academia and the larger society in the triple-helix model [24], which similarly constitutes a shift away from linear models of the science–society relationship.

Note that the three models are ideal types; they are designed to help guide our theorizing. In that sense, they are not mutually exclusive—that is, they can (and frequently do) coexist. Each model corresponds to a class of strategies and techniques associated with managing the science–policy interface that researchers (can) adopt. Each phase thereby follows its own logic [80]. The family of linear models is of particular interest to our research aim. In the following sections we analyse a body of empirical work on the science–policy interface that suggests that the science–policy interface can be described as a gap. Based on an in-depth analysis of this literature, we argue that this diagnosis builds upon an ultimately inadequate linear understanding of the science–policy interface.

## Methodology

### Identification of relevant studies

We proceeded according to the following steps: identification of relevant studies, selection of eligible studies based on abstract and keywords, summarizing the results and narrative synthesis. The authors conducted abstract/title searches in electronic databases (Web of Science, Scopus, PubMed) in October 2019 and January 2020 using combinations of the following search terms (Table 1).

**Table 1:** Overview of search terms

Open science	Responsible research and innovation	Uptake	Impact	Information-seeking behaviours	Policy-making	Matthew effect
Open access	RRI	Absorption	Effect	Information-seeking strategies	Policy design	Cumulative advantage
Open data	RRI indicators	Receptivity	Consequence	Information strategies	Policy development	
Open peer review	MoRRI indicators	Capacity to absorb	Influence	Knowledge-seeking	Policy mak*	
Open methodologies	SuperMoRRI	Reception	Leverage	Knowledge-seeking strategies		
Open science outputs	Responsibility	Absorptive capacity	Clout	Research strategies		
Citizen science	Accountability	Knowledge exchange	Enhanc*	Literature research		
		Knowledge transfer		Evidence		
		Participat*		Evidence-based		
		Research utilization				
		Knowledge brok*				
		Policy gap				
		Policy advice				

* is used for truncating search terms, i.e., enhanc* finds instances of "enhance", enhancement", and the like

### Selection procedure

We conducted a literature search based on standardized keyword strings built from the search terms above, producing 491 studies.^2^ The strategy involved identifying relevant literature on research uptake and then eliminating for lack of references to open science principles and practices. After deduplication, retrieved articles were categorized thematically. In total, 73 articles were included in the analysis based on title and abstract. The following inclusion criteria were applied:

- (National and international) studies attempting to understand or improve academic policy advice/research uptake
- (National and international) studies attempting to understand or improve the policy process
- Available in English
- Full text could be obtained
- Peer-reviewed (review article, commentary, editorial, conference paper, research article)—grey literature was excluded
- Any kind of methodology (quantitative, qualitative, review) was eligible.

One important downside of this methodology is that it only identifies contributions that define research uptake as a problem, leaving out studies that employ a different problem definition. Effectively, this means that fields which do influence policy-making but where the mechanisms are taken for granted are possibly absent from this review.

### Summarizing the results

Following the approach in Contandriopoulos et al. [16, p. 453], we combine a summative and an analytical approach, moving from article synopses to syntheses of how knowledge transfer is problematized with respect to open science practices. Whereas narrative analysis was used to focus on problem definitions and contextual factors, a summative approach was employed for describing commonalities and identifying causal mechanisms of knowledge transfer.

## Literature review: how research does (not) inform policy

### The role of evidence in policy

Research into the role of evidence in policy-making constitutes a field of considerable breadth. Interestingly, a large proportion of empirical work into the science–policy interface has been relatively unperturbed by the historical dimensions of the phenomenon, sketched in the preceding section. Barriers to and facilitators of knowledge transfer are a well-recognized research topic [62, p. 735] with a set of established results. The bulk of the work on knowledge transfer pertains to barriers and facilitators with respect to evidence-based policy-making [51, 62, 64, 67]. Academics have strong motivations to give policy advice, in terms of both demonstrating “impact” to funders and making a difference to society [66]. Boswell (2008, 2009) identifies three functions of expert knowledge in policy-making (cited after Holm and Ploug [45, p. 15]): (1) as an instrument to achieve a (given) aim (instrumental), (2) to confer epistemic authority and thereby legitimacy (legitimating function) and (3) to substantiate already formed policy preferences (symbolic function). To illustrate, Christensen [15, p. 293] points out that at least since the end of the Second World War, particular interest of policy-makers accrues to economics, which has begun to bestow more legitimacy in policy advice than other forms of knowledge. This has been variously attributed to the (supposed) role of economics in ensuring prosperity, and in economics bestowing an aura of rationality on decision-making (ibid.).

Even while there is a vast body of literature studying impact, the relative importance of different factors has not been established [40]. With few exceptions, the literature on the topic fails to clearly define or analyse the fundamental concepts (“research uptake”, “impact”, “policy advice”) and the problems that are at stake. Notable counterexamples include works by Weiss [87], Mitton et al. [62]—who build upon Weiss’s model—and Blewden et al. [4], whereas Cairney, along with various collaborators, has published extensively on the second issue [8, 10, 66, 88]. The respective literature can be grouped as follows:

1. Problem diagnosis: empirical evidence for the evidence–policy gap (mostly qualitative, i.e. interviews and surveys)
2. Problem solving: recommendations for researchers on how to “bridge the evidence–policy gap”
3. Critique: the diagnosis of an evidence–policy gap results from a normative problem definition and an analysis based on a rudimentary (common sense) understanding of policy processes.

Here, we focus on the first and second group to distil the common denominator of the empirical work on why research uptake does (not) work. As Oliver and Cairney [66] point out, the advice offered to academics wishing to engage with policy-makers is frequently inconsistent. We would like to add to this the observation that the analyses of the science–policy interface are, if not inconsistent, then frequently uninformative. In fact, while there is now a large body of work documenting barriers to the use of scientific evidence in policy processes, taken at face value, most of the advice for overcoming barriers to uptake appears commonsensical and generic. Most of it uncritically assumes a gap between academia and policy, otherwise known as the evidence–policy gap [19], that “needs bridging” [71], often going so far as “using the exact phrasing” [66, p. 3] to suggest (rather than demonstrate) that their advice will help foster research uptake. This literature further assumes that policy is rarely based on data, and that greater use of evidence will produce better outcomes, an assumption that remains empirically untested [66]. At best, studies recommend process-related improvement (e.g. by reference to increasing transparency) [19]. Empirical work is frequently case-based, without contextualization of the multifaceted processes underlying policy development [64, 67, p. 4].

### Key factors in research uptake: relationships, resources and research skills

Debates about the nature of the problems have spawned various literature engaging with policy advice from empirical and theoretical standpoints. In drafting this section, we predominantly relied on four narrative reviews of barriers and facilitators for the use of evidence by policy-makers [10, 54, 64, 64, 67, 67] that we amended by including empirical studies. Contacts and relationships (social capital) are reported throughout the literature as major facilitators of evidence use [64, 67, p. 7]. According to Oliver [64, 67, p. 4], timing and opportunity are the most important factors, along with (dis)trust and mutual (dis)respect. Policy-makers seek information that is timely, relevant, credible and available [42, p. 7]. Organizational factors such as (lack of) access to scientific results, (lack of) material and personnel resources and managerial support, and inflexible and nontransparent policy processes are mentioned frequently (ibid. 4 f.) Quality, relevance and reliability of research as well as presentation formats act as facilitators (ibid. 6). However, accessible communication of research involves trade-offs: Clear writing makes research more digestible but at increased cost for researchers [60]. Respondents value researchers who exhibit competence (pragmatism)/reputation), integrity (faithful representation of research) and independence (more important to politicians), and benevolence/commitment [65, p. 122]. For research to be effective in policy-making, a fundamental requirement is effective communication (e.g. [17]), a responsibility ascribed to researchers (e.g. [41]). Lack of understanding/awareness of research on the part of policy-makers was reported as a barrier (ibid. 6), as were (lack of) personal experience, values and judgements. Respondents scarcely attribute lack of uptake to the policy process itself [10]. Indeed, the literature often bemoans a general lack of reflection on policy processes (ibid.). Research uptake is further enabled/hampered by organizational constraints, influence of fads and trends on the policy process [87], corruption and ideology as well as cultural beliefs [39]. The most frequent organizational barriers to research uptake were limited resources (financial or personnel), time constraints (to make decisions or participate in training), high staff turnover and institutional resistance towards change [20]. On the other hand, decision-makers’ willingness to create a culture of knowledge translation and to invest resources was mentioned as a facilitator.

In what follows, we identify key factors that the literature holds (not) to be conducive to research uptake: (i) quality of relationships and informants, (ii) resources and access to research, (iii) communication formats and policy-makers’ research skills, and (iv) the policy context, and discrepancies in values, and goals. As we will demonstrate, the value proposition of open science directly or indirectly relates to several of these factors, which suggests that the genericity of the analysis of barriers carries over to the proposition that open science will enhance research uptake.

(i) *Relationship quality and quality of informants* Relationship quality is a well-recognized research area. Collaboration between researchers and policy-makers, along with relationships and skills, are the most frequently reported facilitators of research uptake [64, 67]. (Long-term) collaboration starting in the early stages of knowledge production is favoured by researchers and policy-makers alike [14]. Mutual mistrust is a well-researched barrier [13, 23, 34, 35, 41]. Researchers are advised to build better communication channels and relationships with policy-makers [31, 34, 38, 42]. While policy-makers worry about bias in research, researchers qualify policy processes as biased [35]. Positivism is thereby an artefact of requests for unbiased truth. The strategies employed by researchers to influence policy are likewise value-laden and cannot be understood solely as evidence-based [9]. Because researchers and policy-makers belong to different communities [55], the role of the knowledge broker has gained importance [28] in facilitating knowledge transfer [32]. Sustained dialogue between researchers and policy-makers is essential for the development of researchers’ perspectives, in-depth knowledge of the policy process, and credibility [31]. This aspect of the problem mirrors the constraints posed by differences in timescales [4, 11, 13, 18, 22]. The prevalence of informal contacts entails that science–policy interactions lack transparency [44]. Policy-makers treat scientific input as an internal concern, with the effect that recommendations by committees remain invisible. Oliver et al. [64, 67] document an increasing amount of research stressing the serendipitous nature of the policy process which gives primacy to informal contacts. In these environments, formalized advice through contract research does not promote transparency, but shifting to research programmes has boosted transparency regarding beneficiary institutions, funding amounts, topics and publication of results [44].

Policy-makers’ advisors reside either inside or outside public bodies [15, p. 295]. The current knowledge transfer landscape includes a set of (more or less) formalized roles [63, p. 3]. Policy-makers trust government sources as well as advocacy, industry and lobby groups, and experts [17, p. 844]. Policy-makers trust their networks and personal contacts most for information [65]; academics are rarely represented in them [65, p. 122]. As their research awareness is low, policy-makers prefer opinion leaders as information sources. Few academics participate directly in the decision-making process (ibid.). Policy-makers prefer local experts, governmental agencies and websites to academic publications. Policy-makers predominantly seek (quantitative) data and statistics [17, p. 842], but also use other information which they consider relevant and timely [64, 67].

(ii) *Organizational factors and access to academic resources* Lack of resources is a frequent barrier to academic policy advice [10, p. 400]. Resources are invested to the extent knowledge exchange is deemed profitable [16, p. 462]. Researchers tend to expect knowledge transfer to produce immediate results [4]. However, the temporal structure of policy-making is ill-attuned to academic influence [48, p. 205], as timescales of policy-making are shorter than those of academia [42]. Time constraints keep policy-makers from directly engaging with research. Timely access to good-quality research is conducive to uptake; poor access and lack of timely research output are frequent barriers [64, 67], as is the short-term nature of research funding [23, p. 467]. Knowledge transfer is deeply embedded in organizational, institutional and policy contexts [16, p. 468] which influence how relationships between academia and government evolve [42, p. 7], but is not featured in tenure/promotion criteria [35]. Patterns of evidence use and management vary across domains and across organizational types [43].

Access to information is important in research uptake [64, 67]. Policy-makers need relevant research to make well-informed decisions [12]. Costs associated with access inhibit research uptake, and public servants use their university affiliations to circumvent this [63]. Research needs to be both accessible (to potential users) and acceptable (in terms of the evidence provided) [60, p. 303]. Accessibility enables timely use of evidence; acceptability can mean scientific acceptability (valid methods, unbiased results, modelling assumptions), institutional acceptability (evidence meets the institutional needs of the decision-maker) or ethical acceptability [60]. There are trade-offs between the accessibility and the acceptability of research findings such that the use of a statistical apparatus might improve the acceptability of a certain evidence base, but only at the cost of its accessibility to nonexperts. External funding may similarly increase accessibility but harm scientific acceptability [60]. The propensity of organizations for research uptake depends on formal and informational structures for organizational learning [1]. Translation via up-to-date research syntheses that are easier to consume and less likely to be biased could help [38]. Systematic reviews are regarded as fundamental in transferring evidence from medical and health research to health policy-making [1, 58, 83], but even systematic reviews require translation [39], making for the importance of intermediaries [34]. Formal structures within research-performing institutions along with mechanisms to make syntheses available could facilitate research uptake [1, 34]. Given policy-makers’ preference for personal contacts, the availability and accessibility of scholarly publications is of secondary concern [65].

(iii) *Communication formats and research skills* Scholarly communication via peer-reviewed publications is ill-attuned to the needs of policy-makers who prefer personal contacts [41]. Potential experts are identified based on engagement with literature, through conferences, personal networks and reputation (e.g. past committee memberships), media presence, and sometimes through self-identification [17, 41]. Oral forms of communication are more commonly used than written material; the ability to communicate clearly and concisely is highly sought after. Policy-makers prefer personal contacts; formal procedures to identify experts are rare [65].

Policy-makers involve such heterogeneous actors as politicians, public servants, administrators, lobbyists and interest groups [76]. Evidence helps decision-makers reduce uncertainty, but policy-makers rely on beliefs and emotions in choosing a problem interpretation [10]. Policy-makers’ abilities in finding and making sense of evidence facilitate research uptake [12, 64, 67]. Policy-makers struggle with knowledge management and have difficulties appraising research [18; 42, p. 7], in addition to a lack of financial resources, knowledge, attitudes and skills [18]. Because uptake depends on data interpretation and analysis skills, mere access to data and other research outputs (systematic reviews, individual studies, grey literature) is not sufficient [53].

(iv) *Policy context and discrepancies in norms and goals* The policy context is fundamental for the use of evidence [4, 16, 31, 49]. Policy-making is an unpredictable, long-term, multilevel process involving networks of policy-makers, paradigms and norms in a quick succession of priorities [10, p. 400; 11, p. 544]. The inclusion of academics and interest groups in the policy process is subject to cultural differences [44]. Research needs to be policy-relevant in the first place to be considered by policy-makers [74], but this is only a necessary (not a sufficient) condition. Policy-making and academia have different goals and success criteria [45, p. 8] Policy is not driven by neutral scientific evidence. Policy-makers are motivated by factors other than research evidence [43, p. 474]. The policy process is inherently normative, involving interests and power relations and necessarily depending on policy-makers’ preferences, goals and values [48, p. 204; 43, p. 473]. These deliberative aspects are difficult to account for in problem-centred analyses of knowledge transfer [23, p. 467]. Evidence pertains to ends and means [43, p. 473], and needs to be embedded in action proposals [16, p. 459]. Researchers work with small, clearly defined problems, whereas policy-makers address problems holistically [48, p. 204]—discrepancies that make collaboration prone to conflict (e.g. [13]). Collaboration is not neutral; it works best when research goals match policy aims [6]. Discrepancies in norms and values influence how the potential for research uptake is perceived [59]; internal validity of information does not by itself influence the use of information [16, p. 457]. Research uptake therefore depends upon relevance to a given policy context [87], legitimacy (of knowledge producers) and accessibility [16, p. 460]. Even where the impact of scientific evidence on policy advice is evident (e.g. [15]), it is not clear whether changes in the culture of policy advice have an impact on policies. The same can be said for research more generally. In addition to having to answer questions of implementation, policy-makers need to worry about being re-elected and striking compromise between competing groups. All these factors limit the extent to which policies can be evidence-based.

## Discussion

In an attempt to interpret the claims of the reviewed literature, we propose considering the following three analytical points. We start with Cairney and collaborators, who argue that the literature on the evidence–policy gap lacks the conceptual resources to describe how policy processes work. If Cairney et al.’s [10] analysis is correct, a deeper understanding of the way policy processes work is necessary to understand how policy advice benefits decision-making. However, instead of attempting to understand policy processes, "researchers have directed their attention at how to increase their own outputs, rather than on understanding the processes behind policy change" [67]. Consequently, the mechanisms by which scientific advice may benefit policy processes are rarely explicated. Even though policy models largely remain implicit in the reviewed literature, we argue that most studies rely on a linear model of the science–policy interface. The linear model frames scientists as producers of expert knowledge and identifies the main challenge in effective forms of communication that deliver the required knowledge to policy-makers. This linear model corresponds to representative democracy [77], suggesting that policy-making takes place primarily in (national, regional, municipal) parliaments and effectively boils down to decisions made by elected representatives of these assemblies.

In light of the contemporary literature, linear conceptions of the science–policy interface are rather outdated. Such linear models dominating the postwar era were superseded by interactive approaches [80]. Today, however, science–policy models seek to include a broad set of stakeholders and civil society actors in policy deliberation. Such type of knowledge integration in the policy domain, however, draws on participatory democracy and consequently involves policy arenas beyond legislative assemblies [33]. This conceptual consideration of the literature on the science–policy interface is crucially important, as it points to the conspicuous absence of interactive and deliberative approaches to policy-making in the reviewed literature. The conceptual preference for the linear model not only impacts on deficient communication as the main problem, but it also entails preferred ways to overcome the shortcomings identified above.

Along the lines of policy advice for actors of representative democracy, Gollust et al. [35] point out that scientists and policy-makers live in different worlds and often do not understand or appreciate the specifics (needs, requirements, logics) of the other [13, 35, 41, 45].^3^ Most of the literature on the evidence–policy gap implicitly assumes a “two cultures model”, highlighting the

differences in academic and political “cultures”: language and jargon, longer scientific timescales, low incentives to engage, differing perceptions of scientific knowledge, and the relative need for scientists to challenge evidence (to ensure it is robust) but for policy makers to generate an image of policy certainty and reconcile evidence with well-established beliefs. There is also a perception that policy makers rely on personal experience, ad hoc links with experts, people they know and trust, and simple decision-making techniques and stories rather than the state-of-the-art in scientific research and sophisticated modeling systems (Lomas and Brown 2009, 906). [10, p. 400]

This model turns on an—ultimately untenable—separation of two aspects of policy advice: reducing *uncertainty* [9, 10] versus reducing *ambiguity* and increasing *clarity* [9]. Cairney et al. [10] further point out that a large proportion of the empirical work has focused on a third aspect that concerns improving the flow of information between decision-makers and researchers. The evidence–policy gap has thus been defined in terms of improving the evidence base, better communication of information, improved interaction with policy-makers, better timing, and the use of knowledge brokers [74].

Finally, we argue that the opacity of the policy-making process in the reviewed literature corresponds to the epistemological opacity of the scientific knowledge in question. By and large, the literature presents scientific knowledge as sacrosanct; scientific knowledge offered in the form of policy advice is taken for granted. Once published, the epistemic status (validity, credibility) of scientific knowledge is no longer questioned and can thus be delivered to policy-makers, notwithstanding the fact that knowledge users might require translation. A deeper analysis of the tenets of the literature on research uptake therefore shows that positivist conceptions of knowledge correspond to a specific view of policy-making as parliamentarian decision-making which in turn corresponds to a linear model of the science–policy interface. Seen from this point of view, scientific knowledge is sought after because of its desired capacity to solve policy problems. Likewise, influential interpretations of open science as the provision of open access to scientific outputs are indicative in omitting interpretations that foreground the significance of knowledge creation (public participation and engagement).

It is once more interesting to note the absence of alternative approaches in the reviewed literature. For instance, the literature on the evidence–policy gap never speaks to constructivist epistemologies (knowledge as co-constructed by researchers, policy-makers and civil society actors). Equally, the epistemic role of users, patient groups, (health) professionals or communities of practice is rarely touched upon. Epistemologies corresponding to participatory models of policy deliberation understand scientific knowledge as co-constructed, context-dependent and situated. It follows that for knowledge to be policy-relevant and effectively implemented, it matters that it is created in a participatory form.

As we have attempted to show, the absence of contributions that ostensibly draw on participatory policy models and related forms of knowledge production is indicative of the implicit assumptions in the reviewed literature on the evidence–policy gap.

## Conclusion

Scientific policy advice is essential for policy-makers, both in terms of addressing immediate issues and in terms of long-term planning. Based on a literature review, we investigated the role of open science and its potential to enhance the uptake of scientific knowledge in the policy domain. Our main findings are as follows:

1. Our analysis identified a promissory discourse suggesting the potentials of open science (e.g. [63, 82]). Using a hypothetical language, assumptions are voiced that open science *might*, *would* or *could* increase the uptake of scientific knowledge in the policy domain, however without the presentation of evidence to substantiate such optimistic claims as yet.
2. We found that a surprisingly small body of literature on the uptake of scientific knowledge in the policy domain *explicitly* addresses the role of open science practices in this regard.
3. By contrast, we found a fairly developed discourse in the medical literature that recognizes a low uptake of scientific knowledge in the policy domain and frames this problem as “evidence–policy gap”. This literature concludes that:
  (i) Scientists and policy-makers live in different worlds and accordingly pursue incommensurable logics.
  (ii) The improvement of communication skills on the part of scientists would be effective in bridging the evidence–policy gap.
  (iii) The literature on the evidence–policy gap predominantly draws on the linear model of the science–policy interface, a conception then criticized as rather outdated [80].
4. Finally, we found a glaring absence of engagement with newer literature on the integration of scientific knowledge in the policy domain. By that we refer to conceptions of the science–policy interface that point to the importance of deliberative and participatory practices. The recognition of newer approaches such as responsible research and innovation were equally absent in the reviewed literature.

In light of these findings, we conclude that the potential for open science practices to bridge the observed evidence–policy gap has as yet not been demonstrated. Accordingly, the claim that open science can boost the utilization of publicly available scientific results and data by policy-makers is empirically largely unsubstantiated. Critical observers noted that accessibility of scientific literature is neither necessary nor sufficient [68, 78]. What matters instead is how a more open, participatory form of research affects scientific knowledge production in the first place. These rather sobering findings raise further questions with respect to modelling the science–policy interface. As we have argued, expectations to the effect that open science will increase research uptake are often linked to a linear understanding of knowledge transfer.

Against this backdrop, we point to the relevance of more recent literature on the science–policy interface. In particular, the consultation of a broader set of stakeholders has been suggested as a more inclusive and effective form of knowledge integration in the policy domain [80]. Future attempts to utilize the potentials of open science may therefore benefit from turning to deliberative forms of policy advice. In such a way, open science may have more to offer than what is now conceived almost exclusively as the provision of access to scientific outputs and more effective scholarly communication. This potential is even greater where participation is made possible from the outset of knowledge-making processes (upstream engagement). Indeed, participatory approaches and the contribution of more inclusive forms of knowledge-making have seen some success [27, 33]. However, these efforts were not framed in terms of open science, but rather as deliberative democracy [50], participatory technology assessment [5], public engagement [3], or responsible research and innovation [68], respectively. A stronger mutual recognition of these as yet separate bodies of literature may help to advance the integration of scientific knowledge in the policy domain.

## Data Availability

This contribution is based upon a literature review. The materials used are therefore available subject to copyright agreements between the authors of these publications and their publishers.

## Footnotes

1 The term “democratic school” is ambiguous in this context, since Fecher and Friesike refer to access to scientific knowledge without having a specific user in mind, rather addressing a generic subject of knowledge ([25, p. 25]. Knowledge provided is not appropriated by nonscientific audiences and therefore boils down to a science-to-science communication of research outputs.

2 For a full list of search strings, databases searched and search results, see Reichmann et al. [73], p. 93 ff.

3 Choi et al. [13] argue in a similar vein that researchers and policy-makers differ substantially in their goals, values, attitudes towards information, languages, perceptions of time, and career paths (e.g. [13]. If correct, this entails that researchers need to balance conflicting roles and identities when engaging with policy-makers in policy contexts. These have been described with reference to Mertonian disinterestedness [61] as an attitude associated with argumentative rigour and rationality which bears the associated risk of policy irrelevance [40].
